# Dissecting Genetic Control of HLA-DPB1 Expression and Its Relation to Structural Mismatch Models in Hematopoietic Stem Cell Transplantation

**DOI:** 10.3389/fimmu.2018.02236

**Published:** 2018-10-05

**Authors:** Thuja Meurer, Esteban Arrieta-Bolaños, Maximilian Metzing, Mona-May Langer, Peter van Balen, J. H. Frederik Falkenburg, Dietrich W. Beelen, Peter A. Horn, Katharina Fleischhauer, Pietro Crivello

**Affiliations:** ^1^Institute for Experimental Cellular Therapy, University Hospital Essen, Essen, Germany; ^2^Institute for Transfusion Medicine, University Hospital Essen, Essen, Germany; ^3^Department of Hematology, Leiden University Medical Center, Leiden, Netherlands; ^4^Department of Bone Marrow Transplantation, West German Cancer Center, University Hospital Essen, Essen, Germany; ^5^Deusches Konsortium für Translationale Krebsforschung (DKTK), Heidelberg, Germany

**Keywords:** HLA-DPB1, expression levels, SNP, rs9277534, hematopoietic stem cell transplantation, high risk non-permissive mismatches, T cell epitope, T cell alloreactivity

## Abstract

HLA expression levels have been suggested to be genetically controlled by single nucleotide polymorphisms (SNP) in the untranslated regions (UTR), and expression variants have been associated with the outcome of chronic viral infection and hematopoietic stem cell transplantation (HSCT). In particular, the 3′UTR rs9277534-G/A SNP in HLA-DPB1 has been associated with graft-versus-host-disease after HSCT (Expression model); however its relevance in different immune cells and its mode of action have not been systematically addressed. In addition, there is a strong though not complete overlap between the rs9277534-G/A SNP and structural HLA-DPB1 T cell epitope (TCE) groups which have also been associated with HSCT outcome (TCE Structural model). Here we confirm and extend previous findings of significantly higher HLA-DPB1 expression in B cell lines, unstimulated primary B cells, and monocytes homozygous for rs9277534-G compared to those homozygous for rs9277534-A. However, these differences were abrogated by interferon-γ stimulation or differentiation into dendritic cells. We identify at least seven 3′UTR rs9277534-G/A haplotypes differing by a total of 37 SNP, also characterized by linkage to length variants of a short tandem repeat (STR) in intron 2 and TCE group assignment. 3′UTR mapping did not show any significant differences in post-transcriptional regulation assessed by luciferase assays between two representative rs9277534-G/A haplotypes for any of eight overlapping fragments. Moreover, no evidence for alternative splicing associated with the intron 2 STR was obtained by RT-PCR. In an exemplary cohort of 379 HLA-DPB1 mismatched donor-recipient pairs, risk prediction by the Expression model and the Structural TCE model was 36.7% concordant, with the majority of discordances due to non-applicability of the Expression model. HLA-DPB1 from different TCE groups expressed in the absence of the 3′UTR at similar levels by transfected HeLa cells elicited significantly different mean alloreactive CD4+ T-cell responses, as assessed by CD137 upregulation assays in 178 independent cultures. Taken together, our data provide new insights into the cell type-specific and mechanistic basis of the association between the rs9277534-G/A SNP and HLA-DPB1 expression, and show that, despite partial overlap between both models in HSCT risk-prediction, differential alloreactivity determined by the TCE structural model occurs independently from HLA-DPB1 differential expression.

## Introduction

Genetic control of HLA expression is a topic of increasing interest due to accumulating evidence for its relevance in different biomedical areas including infectious disease and transplantation. In particular, expression levels of HLA-A, -C and -DPB1 genes have been shown to be associated with specific single nucleotide polymorphism (SNP) variation in the untranslated regions (UTR) (1–5). The presence of high-expression variants was in turn associated with poor clearance of chronic viral infections including HIV (1, 6) and HBV (5), suggesting mechanistically increased natural killer (NK) cell inhibition and higher T cell tolerance, respectively. Interest in HLA expression was further fostered by the observation that donor-recipient HLA mismatches involving a high-expression variant in the patient are associated with the risks of developing graft-versus-host-disease (GvHD) after Hematopoietic Stem Cell Transplantation (HSCT), both for HLA-C (7) and HLA-DPB1 (8). The latter involves the bi-allelic SNP rs9277534, with the G-variant leading to high expression and the A-variant to low expression.

The mechanisms underlying the above-mentioned associations between SNP variation and HLA expression are only partly elucidated. For HLA-A, they have been suggested to be correlated with differential methylation and/or the usage of different polyadenylation sites (PAS) (2, 3). For HLA-C and HLA-DPB1, no specific mechanisms for the observed genetic control of expression levels have been postulated to date. Moreover, the association of the rs9277534-G/A SNP with HLA-DPB1 expression has been demonstrated only in a limited set of immune cells of the B cell lineage (5, 8).

Two conceptually different models of HLA-DPB1 mismatches associated with favorable or less favorable outcome of unrelated HSCT have been developed in recent years, and have been validated in independent clinical studies (8–14). The Expression model is based on the assumption that patient-specific HLA-DP antigens with high levels of cell surface expression could be recognized more efficiently by alloreactive donor T cells than patient-specific HLA-DP antigens with low levels of cell surface expression. Therefore, genetic association of HLA-DPB1 expression with the bi-allelic rs9277534 SNP in the 3′UTR is used to assign high or low expression levels to alleles carrying the G- or the A-variant, respectively (5). The TCE Structural model considers variability in the HLA-DPB1 coding sequence translating into polymorphisms in the peptide binding domain, which in turn leads to the generation of structural epitopes recognized by alloreactive T cells. Based on this, at least three different TCE groups were identified, each comprising different HLA-DPB1 alleles sharing the relevant structural epitope. TCE group assignment of HLA-DPB1 alleles was defined either by cross-reactivity of alloreactive T cells (9) or, more recently, by the combined median impact of polymorphic amino acids on T-cell alloreactivity, termed functional distance (FD) (15). The three TCE groups are not equivalent but follow a hierarchical order of immunogenicity, with TCE group 1 > TCE group 2 > TCE group 3. Interestingly, due to strong linkage disequilibrium (LD) between the HLA-DPB1 3′UTR and its coding sequence, there is a strong though not complete overlap between HLA-DPB1 alleles carrying the rs9277534 SNP and the structural TCE groups (16). The relationship between the rs9277534 SNP Expression model and the TCE structural mismatch model for high-risk non-permissive HLA-DPB1 mismatches in HSCT is currently unknown.

In this study, we have set out to fill these gaps by (i) investigating rs9277534 SNP association with transcriptional and cell surface HLA-DPB1 expression in different Antigen Presenting Cells (APC); (ii) 3′UTR haplotype mapping of different HLA-DPB1 alleles and functional testing of their regulatory activity; (iii) comparing the demographics of risk prediction based on the Expression model and the TCE Structural mismatch model for HLA-DPB1 in an exemplary cohort of unrelated HSCT; and (iv) assessment of *in vitro* T cell alloreactivity against different HLA-DPB1 TCE groups at similar transcriptional expression levels in transfected APC.

## Materials and methods

### Cells and cell lines

Peripheral blood mononuclear cells (PBMC) were obtained from healthy blood donors from the University Hospital Essen after informed consent under Ethical Review Board approval, in accordance with the Declaration of Helsinki. EBV-transformed B lymphoblastoid cell lines (BLCL) were generated from PBMC by standard procedures (17), or purchased from the European Collection of Authenticated Cell Cultures (ECACC). HLA-DPB1 typing of the healthy donors was performed by sequence-specific oligonucleotide probing (LABType SSO, One Lambda, Canoga Park, CA, USA) according to the manufacturer's recommendations, under accreditation by the European Federation for Immunogenetics. A list of PBMC and BLCL used in this study and their HLA-DPB1 types is presented in Tables 1, 2. Typing of the rs9277534 SNP was performed by sequence-specific primer (SSP) PCR (Table 3), and confirmed by Sanger sequencing of the 3′UTR following published methods (5).

**Table 1:** BLCL used in this study.

**BLCL^a^**	HLA-DPB1^*^			**Application in this study^d^**
	**Allele**	**rs9277534^b^**	**3′UTR Haplotype^c^**	
MGAR	04:01	AA	6	Exp, Hap, Luc
MOU	02:01	AA	6	Exp, Hap, Luc
SWEIG007	04:02	AA	6	Exp, Hap
*OSR-6924*	*02:01, 04:02*	AA	6	Exp, Luc
*OSR-2674*	*04:01, 17:01*	AA	6	Exp
*OSR-7891*	*04:01, 17:01*	AA	6	Exp
*OSR-5678*	*02:02, 04:01*	AA	6	Exp
*OSR-1629*	*02:01*	AA	6	Exp
*OSR-2674*	*04:01, 17:01*	AA	6	Exp
BM21	10:01	GG	1	Exp, Hap, Luc
APA	05:01	GG	1	Exp, Hap, Luc
BEL7MON	03:01	GG	1	Exp, Luc
AKIBA	09:01	GG	1	Exp, Hap
KAS116	13:01	GG	3	Exp, Hap
PLH	15:01	GG	1	Exp, Hap
SLE005	03:01	GG	1	Exp, Hap
VAVY	01:01	GG	2	Exp, Hap
H0301	05:01	GG	1	Exp

a *BLCL were purchased from the ECACC (regular font), or generated locally (italic)*.

b *rs9277534-G/A as determined locally*.

c *3′UTR haplotypes refer to those identified in Table 5*.

d *Use in this study for quantification of HLA-DPB1 protein and transcript expression (Exp), 3′UTR haplotype sequencing (Hap), or Luciferase assays (Luc)*.

**Table 2:** PBMC used in this study.

	HLA-DPB1^*^			**Application in this study^d^**
**PBMC^a^**	**Allele^a^**	**rs9277534^b^**	**3′UTR Haplotype^c^**	
UKE-9117	04:01, 04:02	AA	6	Exp(B,Mono,DC), T-cell culture
UKE-9149-04	02:01, 04:01	AA	6	Exp(B,Mono,DC), T-cell culture
UKE-9169-02	02:01, 04:01	AA	6	Exp(B,Mono,DC), T-cell culture
UKE-9169-03	04:01, 04:02	AA	6	Exp(B,Mono,DC), T-cell culture
UKE-9731	02:01, 04:01	AA	6	Exp(B,Mono,DC), T-cell culture
UKE-11103	02:01, 04:01	AA	6	Exp(B,Mono,DC), T-cell culture
UKE-11154	02:01, 04:01	AA	6	Exp(B,Mono,DC), T-cell culture
UKE-9068	02:01, 04:02	AA	6	Exp(B,Mono,DC)
UKE-9765	04:01, 04:02	AA	6	Exp(B,Mono,DC)
UKE-11116	02:01, 04:01	AA	6	Exp(B,Mono,DC)
UKE-9133-01	01:01, 13:01	GG	2, 3	Exp(B,Mono,DC)
UKE-9157	01:01, 03:01	GG	1, 2	Exp(B,Mono,DC)
UKE-8305B	01:01, 11:01	GG	2, 3	Exp(B,Mono,DC)
UKE-8097-A	01:01, 03:01	GG	1, 2	Exp(B,Mono,DC)
UKE-020215B	01:01, 03:01	GG	1, 2	Exp(B,Mono,DC)
UKE-8461C	01:01, 03:01	GG	1, 2	Exp(B,Mono,DC)
UKE-11012B	03:01, 13:01	GG	1, 3	Exp(B,Mono,DC)
UKE-11055A	01:01, 13:01	GG	2, 3	Exp(B,Mono,DC)
UKE-11209A	01:01, 03:01	GG	1, 2	Exp(B,Mono,DC)
UKE-11209B	03:01	GG	1	Exp(B,Mono,DC)
UKE-11177	02:01, 04:01	AA	6	Exp(B,Mono)
UKE-7547	03:01	GG	1	Exp(B,Mono)
UKE-8117A	04:01, 04:02	AA	6	T-cell culture
UKE-7595	04:01	AA	6	T-cell culture
UKE-7903-3	04:01, 04:02	AA	6	T-cell culture
UKE-020215A	04:01	AA	6	T-cell culture
UKE-8522C	04:01	AA	6	T-cell culture
UKE-7903-2	02:01, 04:02	AA	6	T-cell culture
UKE-8251C	02:01, 04:01	AA	6	T-cell culture
UKE-8360A	02:01, 04:01	AA	6	T-cell culture
UKE-8522A	02:01, 04:02	AA	6	T-cell culture
UKE-8522B	02:01, 04:02	AA	6	T-cell culture
UKE-10999	04:01	AA	6	T-cell culture
UKE-11012A	04:01, 04:02	AA	6	T-cell culture
UKE-10917	04:01, 04:02	AA	6	T-cell culture
UKE-9595	04:01, 04:02	AA	6	T-cell culture
UKE-9822	04:01	AA	6	T-cell culture
UKE-9110	02:01, 04:01	AA	6	T-cell culture
UKE-7964-2	04:01	AA	6	T-cell culture
UKE-9320B	02:01, 04:01	AA	6	T-cell culture
UKE-10294A	04:01	AA	6	T-cell culture
UKE-7713	03:01, 04:02	AG	1, 6	T-cell culture
UKE-7964-1	03:01, 04:02	AG	1, 6	T-cell culture
UKE-120315	03:01, 04:02	AG	1, 6	T-cell culture
UKE-8360B	03:01, 04:02	AG	1, 6	T-cell culture
UKE-9487	03:01, 04:01	AG	1, 6	T-cell culture
UKE-9751C	03:01, 04:02	AG	1, 6	T-cell culture
UKE-8097B	02:01, 03:01	AG	1, 6	T-cell culture
UKE-8461A	02:01, 03:01	AG	1, 6	T-cell culture
UKE-8360C	04:01, 14:01	AG	1, 6	T-cell culture
UKE-9145	04:01, 14:01	AG	1, 6	T-cell culture

a PBMC were isolated from peripheral blood of 51 healthy individuals. HLA-DPB1

* *allele typing was performed as described in Materials and Methods*.

b *rs9277534-G/A typing was determined by SSP-PCR as described in Materials and Methods*.

c *3′UTR haplotypes were assigned based on the linkage disequilibrium to HLA-DPB1 alleles as reported in Table 4*.

d *PBMC samples were used for quantification of HLA-DPB1 expression (Exp) in B cells (B), monocytes (Mono) and dendritic cells (DC), and/or in functional testing of alloreactive T-cell cultures stimulated with HeLa cells expressing single HLA-DPB1 antigens (T-cell culture)*.

**Table 3:** PCR reactions used in this study.

**Reaction^a^**	**Type^b^**	**Purpose**	**Primers^c^**	**Cycling conditions**	**Efficiency^d^**
rs9277534 SSP-PCR	STD-PCR	rs9277534 SNP typing	FW-A:‘ATCCATTTATGTCTCAGACCA’ (rs9277534-A) FW-G:‘TCCATTTATGTCTCAGACCG’ (rs9277534-G) RV: ‘GGTCCTATCAGGCAGATTTGCAG’ (both)	95°C for 15” 60°C for 30” 72°C for 30”	N.A.
HLA-DPB1 short mRNA	qPCR	Gene expression	FW-a:‘AAGAAAGTTCAACGAGGATCTGC’ (exon 5) RV-b:‘GAAGAAGGGAACATGGTTGGAG’ (exon 6)	95°C for 15” 62°C for 1'	92%
HLA-DPB1 long mRNA	qPCR	Gene expression	FW-c:‘ATGACACTCTTCTGAATTGACTG’ (exon 6) RV-d:‘GGTAATGATAAAACATGCTCTC‘ (exon 6)	95°C for 15” 62°C for 1'	97%
F1^*e*^	STD-PCR	Molecular cloning	FW-F1A:‘ACAGGGTTCCTGAGCTC’ (A-haplotype) RV-F1A:‘TTGGAAGTTGAAGGTCTGTC’ (A-haplotype) FW-F1G:‘ACAGGGTTCCTGACCTC’ (G-haplotype) RV-F1G:‘TGGGAAGCTGAGGGTC’ (G-haplotype)	95°C for 15” 63°C for 30” 72°C for 30”	N.A.
F2^*e*^	STD-PCR	Molecular cloning	FW-F2A:‘TCCAGGACAGACCTTCAAC’ (A-haplotype) FW-F2G:‘TCCAGGACAGACCCTCAG’ (G-haplotype) RV-F2:‘GAAACAGTGCTTTGAATCAAAGAGC’ (both)		
F3^*e*^	STD-PCR	Molecular cloning	FW-F3:‘GCTCTTTGATTCAAAGCACTG’ (both) RV-F3A: ‘AAACAAACAGTCATGTTGGG‘ (A-haplotype) RV-F3G:‘AAACAAACACTTATGTTGGGTTTTG’ (G-haplotype)		
F4^*e*^	STD-PCR	Molecular cloning	FW-F4A:‘CAAAACCCAACATGACTGTTTG’ (A-haplotype) RV-F4A:'TTATTAACTCCTACTGTCTACTAAAACC‘ (A-haplotype) FW-F4G:'CAAAACCCAACATAAGTGTTTG' (G-haplotype) RV-F4G:'TTATTAACTCCTACTGTTTACTAAAACCC‘ (G-haplotype)		
F5^*e*^	STD-PCR	Molecular cloning	FW-F5A:‘TTTAGTAGACAGTAGGAGTTAATAAAGAAG' (A-haplotype) RV-F5A:'CCATTATACAATAGTTAACATATCCCC‘ (A-haplotype) FW-F5G: 'TTTAGTAAACAGTAGGAGTTAATAAAGAAG' (G-haplotype) RV-F5G:' ACATTATACAATAGTTAACATATCTCCAC' (G-haplotype)		
F6^*e*^	STD-PCR	Molecular cloning	FW-F6A:‘GGATATGTTAACTATTGTATAATGGGG' (A-haplotype) FW-F6G:'AGATATGTTAACTATTGTATAATGTGGC' (G-haplotype) RV-F6:‘GAGGGCACTAAACTTGATTTG' (both)	95°C for 15” 63°C for 30” 72°C for 30”	N.A.
F7^*e*^	STD-PCR	Molecular cloning	FW-F7:‘CCCCCAAATCAAGTTTAGTG' (both) RV-F7A:‘TGGGTCCTATCAGGCAG' (A-haplotype) RV-F7G:‘CGGGTCCTATCAGGC' (G-haplotype)		
F8^*e*^	STD-PCR	Molecular cloning	FW-F8:‘CTGCAAATCTGCCTGATAG' (both) RV-F8: ‘TTCATTTAACTTCTTAATGGTAATGATAAAAC’ (both)		
HLA-DPB1 exon 2-4 splicing	RT-PCR	Alternative splicing	FW-e:‘GCTTCCTGGAGAGATACATC’ (exon 2) RV-f:‘CAGCTCGTAGTTGTGTCTGC’ (exon 2) RV-g:‘TTGAATGCTGCCTGGGTAG’ (exon 3) RV-h: ‘AGCTCCCGTCAATGTCTTAC’ (exon 4)	95°C for 1' 55°C for 30” 72°C for 1'	N.A.
HLA-DPB1 exon 2	qPCR	Gene expression	FW-e:‘GCTTCCTGGAGAGATACATC’ (exon 2) RV-f:'CAGCTCGTAGTTGTGTCTGC' (exon 2)	95°C for 15” 62°C for 1'	100%

a *Reactions are reported in order of appearance*.

b *STD-PCR: standard PCR performed on genomic or plasmid DNA. qPCR: quantitative real-time PCR performed on cDNA obtained as described in Materials and Methods. RT-PCR: reverse transcriptase PCR performed on cDNA as described in Materials and Methods. STD-PCR and RT-PCR were performed using Amplitaq GOLD DNA polymerase and buffer (ThermoFisher Scientific), qPCR were performed using SYBR Green Real-time Mastermix (ThermoFisher Scientific)*.

c *For each primer, name, sequence, and target region or polymorphism in HLA-DPB1 are reported. FW: forward primer; RV: reverse primer*.

d *Efficiency of qPCR reactions was calculated by quantifying the target template in 5-fold serial dilution of cDNA for 10 ng to 16 pg. All qPCR reactions resulted in high reaction efficiency comparable to the qPCR targeting GAPDH as reference gene (Efficiency = 93%) (5)*.

e *F1-F8 fragments of the HLA-DPB1 3′UTR from A- or G-Haplotype were amplified by PCR and subsequently cloned downstream of luciferase gene Luc2 in pmirGLO vector (see also Figure 5). The following restriction sites were added at the 5' of each primer: NheI for forward primers and SalI for reverse primers*.

Stable single HLA-DP HeLa cells transfectants (HeLa-II) were generated by retroviral gene transfer of HLA-DPB1 and DPA1 together with the invariant chain (Ii), HLA-DM, and CD80 as previously described (18). Monocyte-derived dendritic cells (DC) were obtained from frozen PBMC after MicroBead sorting of CD14+ cells (Miltenyi Biotec, Bergisch Gladbach, Germany) and incubation with 500 IU/mL Interleukin (IL)-4 (R&D systems, Minneapolis, MN, USA) and 1000 IU/mL Granulocyte Macrophage Colony-Stimulating Factors (GM-CSF; Miltenyi Biotec, Bergisch Gladbach, Germany) for 5 days. Immature and mature DC were subsequently obtained by incubation with medium alone or in the presence of 10 ng/mL IL-1β (Miltenyi Biotec, Bergisch Gladbach, Germany), 10 ng/mL tumor necrosis factor (TNF)-α (Miltenyi Biotec, Bergisch Gladbach, Germany), 1000 IU/mL IL-6 (Miltenyi Biotec, Bergisch Gladbach, Germany), and 1 μg/mL prostaglandin E2 (PGE2) (Sigma Aldrich, St.Louis, MO, USA) for 24 h, respectively, as described (19). Immature and mature DC were discriminated by the presence or absence of CD83 within the DC population identified as positive for CD11c and negative for CD14 (20).

### Monoclonal antibodies (mAb) and flow cytometry

The following mAb were used: CD3 krome orange [clone UCHT1] (Beckmann Coulter, Brea, CA, USA), CD4 phycoerythrin-cyanin 7 (PE-Cy7) [clone SK3] (BD Bioscience, Franklin Lakes, NJ, USA), CD8 pacific blue [clone B9.11] (Beckmann Coulter), CD11c PE [clone S-HCL3] (BD Bioscience), CD14 fluorescein isothiocyanate (FITC) [clone MϕP9] (BD Bioscience), CD19 allophycocyanin [clone HIB19] (BD Bioscience), CD137 allophycocyanin [clone 4B4-1] (BD Bioscience), CD83 PE [clone HB15] (Miltenyi Biotec), HLA-DP PE [clone B7/21] (21) (Leinco Technologies, Inc. St.Louis, MO, USA), HLA-DR PE [clone L243] (BD Bioscience), murine IgG2A PE [clone S43.10] (Miltenyi Biotec) and murine IgG3 PE [clone A112-3] (BD Bioscience). Flow cytometric determinations were performed on a Gallios™ 10/3 cytometer (Beckman Coulter, Brea, CA, USA), using the Kaluza for Gallios Acquisition software (Version 1.0, Beckman Coulter) and the data were analyzed with Kaluza Analysis Software (Version 1.3, Beckman Coulter).

### Quantification of HLA-DP and DR cell surface expression levels

HLA-DP and HLA-DR cell surface expression levels were quantified by flow cytometry staining by converting the median fluorescence intensity (MFI) observed with the relevant mAb into Molecules of Equivalent Soluble PE (MEPE) (22), using Sphero Rainbow Calibration Particles (Spherotech, Lake Forest, IL, USA) according to the manufacturer's recommendations. MEPE values were corrected for non-specific binding by subtracting the corresponding fluorescent background detected with isotype controls. Gating strategies were as follows: BLCL were analyzed as homogenous population in forward and side scatter dot plots; primary B cells and monocytes were gated in total PBMC as negative for CD4 and CD8 but positive for CD19 or CD14, respectively, before or after 48 h incubation with 200 IU/ml IFN-γ (Axxora, Farmingdale, NY, USA); immature and mature DC were analyzed by gating cells negative for CD14 and positive for CD11c after *in-vitro* differentiation.

### Quantification of HLA-DPB1 transcript levels

HLA-DPB1 transcript levels were quantified from reverse transcribed cDNA by quantitative PCR (qPCR). Total RNA was extracted from 0.5–5 × 10^6^ cells using the PureLink RNA mini kit (ThermoFisher Scientific, Waltham, MA, USA), and cDNA was synthetized from 0.5 to 2 μg total RNA with the High Capacity cDNA Reverse Transcription Kit (ThermoFisher Scientific). qPCR reactions were designed based on SYBR Green chemistry (ThermoFisher Scientific) using a previously described qPCR for GAPDH (5) as normalizer. The normalized amount of HLA-DPB1 mRNA was expressed as 2^−deltaCt^ with delta Ct = Ct_HLA_-DPB1 −Ct_GAPDH_. qPCR primers, conditions and characteristics are shown in Table 3.

### Identification of HLA-DPB1 3′UTR haplotypes

HLA-DPB1 3′UTR nucleotide sequences were aligned from the IMGT/HLA database release 3.31.0 (2018-01) (23). Haplotypes were assigned according to polymorphisms located in the first 671 bp of the transcribed 3′UTR, i.e., the last 4 bp of exon 5 and the first 667 bp of exon 6. The nucleotide sequence of selected haplotypes was confirmed by direct Sanger sequencing (Seqlab, Göttingen, Germany) on both strands of a 667 bp 3′UTR PCR fragment obtained from genomic DNA according to previously described protocols (5).

### Dual luciferase assay

HLA-DPB1 3′UTR fragments or control wild-type (WT) and mutant (mut) target sequence of hsa-miR-21 (mir21-WT and mir21-mut) were pre-amplified by PCR (primers and conditions in Table 3) or synthetized *in vitro* (Eurofins Genomics, Ebersberg, Germany). 3′UTR fragments and controls were cloned into the pmirGLO vector (Promega, Madison, WI, USA) downstream of the luciferase reporter gene (luc2) and transfected into HeLa cells or BLCL by electroporation with the Neon transfection system (Invitrogen, USA), according to the manufacturer's recommendations. Luciferase activity was measured after 24 h with a Dual Luciferase Reporter Assay System (Promega) using the monochromator multimode microplate reader LB 943 Mithras2 (Berthold Technologies, Bad Wildbad, Germany). Luciferase activity under the control of mir21-WT or mir21-mut was used as positive and negative controls, respectively, since the expression of the relevant miRNA hsa-miR-21 was shown to be abundant in both HeLa and BLCL (24, 25). The *Renilla* luciferase gene (hRluc-neo fusion) included in the same vector was used as transfection control for normalization of the luc2 signal.

### Assessment of splicing in HLA-DPB1 mRNA by RT-PCR

Splicing between exons 2–4 of HLA-DPB1 mRNA was assessed by standard RT-PCR on total RNA extracted from BLCL; RNA and reverse-transcribed cDNA were prepared as described above. cDNA samples were used as template for 3 PCR reactions designed to target exon 2 alone, exon 2–3, and exon 2–4 with primers and PCR condition described in Table 3. Analysis of the PCR products was performed using standard agarose gel electrophoresis.

### Alloreactive T-cell cultures

Alloreactive T-cell effectors were raised against individual HLA-DP alloantigens by *in vitro* stimulation of CD4+ T cells with HeLa-II cells. Briefly, CD4+ T cells were isolated from frozen PBMC of HLA-DPB1-typed healthy blood donors (Table 2) by MicroBeads sorting (Miltenyi Biotec, Bergisch Gladbach, Germany), and stimulated for 14 d at a 3:1 ratio with irradiated (100 Gy) HeLa-II expressing the allogeneic HLA-DPB1 allele of interest in culture medium supplemented with 10% heat-inactivated AB human serum (Sigma-Aldrich) and 50 IU/mL IL-2 (Miltenyi Biotec, Bergisch Gladbach, Germany). After 2 weeks, T cells were re-stimulated for 24 h with fresh HeLa-II carrying the relevant allogeneic HLA-DPB1 allele or autologous HLA-DPB1 allele(s), or medium alone. The specific T-cell response was quantified by flow cytometry as the percentage of gated CD4+ T cells upregulating cell surface expression of CD137, corrected for the background response to HeLa-II expressing autologous HLA-DPB1.

### Statistical analysis

Statistical analysis was performed using the Prism 6 software (GraphPad Software, La Jolla, CA, USA), using the two-tailed unpaired *t*-test or the Wilcoxon matched-pairs signed rank Test.

## Results

### High and low cell surface expression of HLA-DP in relation to rs9277534 SNP in different cell types

**Figure 1:**
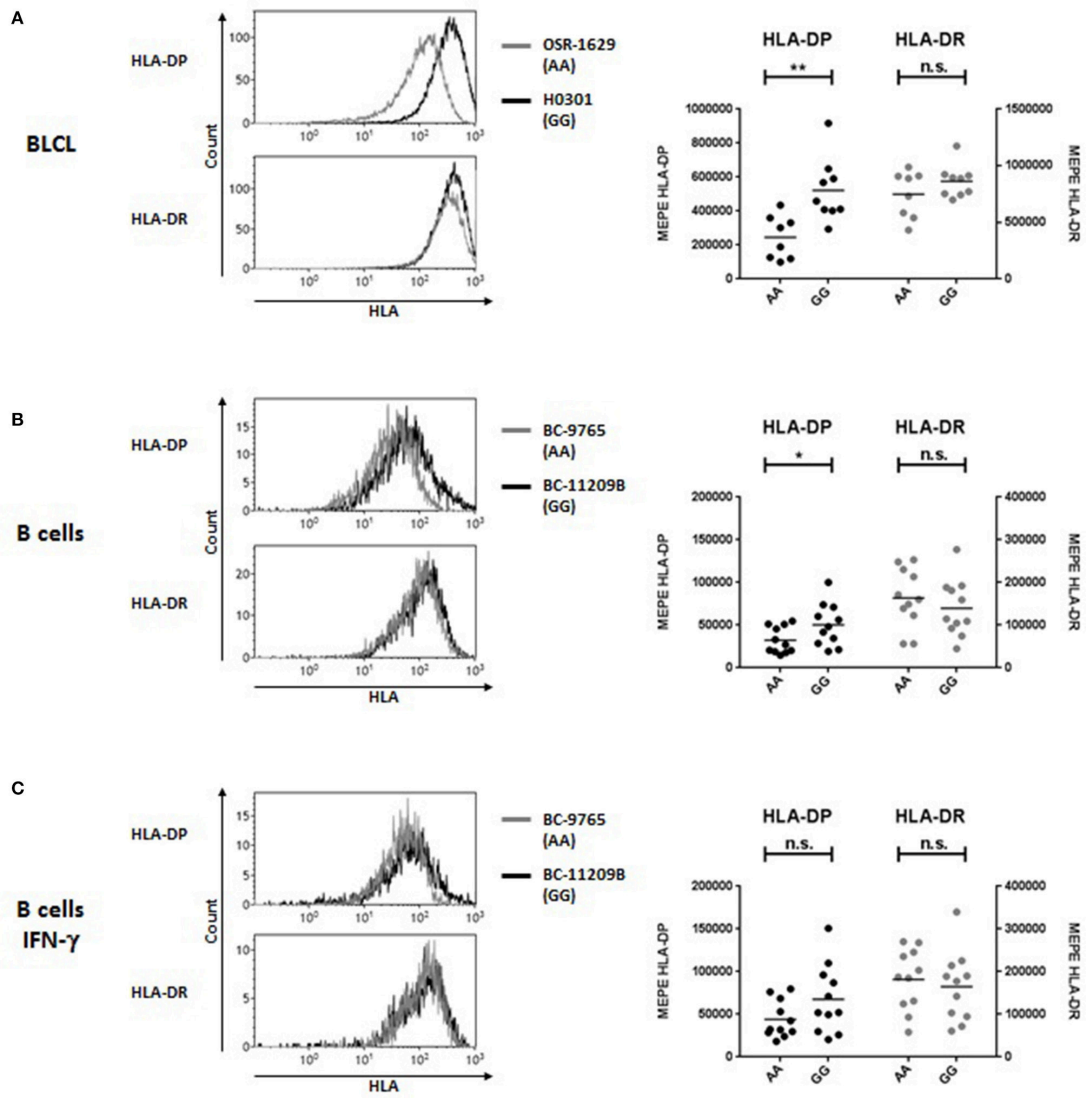
HLA-DP and HLA-DR cell surface expression in relation with rs9277534 SNP in B cells. Cell surface staining for HLA-DP and HLA-DR was performed with the mAb B7/21 and L243, respectively, on **(A)** BLCL (*N* = 17) (**B,C**) CD19+ B cells in PBMC after 48 h incubation with medium (*N* = 22, **B**) or with 200 IU/ml IFN-γ (*N* = 22, **C**). Cells were homozygous for either rs9277534-A/A (AA) or rs9277534-G/G (GG) (Tables 1, 2). Shown are two representative FACS plots (left panel) and the Molecules of Equivalent Soluble PE (MEPE) of all samples (right panels). Statistical comparisons were performed by the two-tailed unpaired *t*-test (n.s. not significant; *p < 0.05; **p < 0.01). Mean ± SD are reported in Table 4.

Association of the bi-allelic rs9277534-G/A SNP variants with high and low HLA-DP expression levels, respectively, was originally reported by Thomas et al. on the cell surface of primary B cells (5) and more recently observed at the transcriptional level in BLCL by Petersdorf and collaborators (8). Here we quantified HLA-DP cell surface expression on BLCL by MEPE flow cytometry, using the mAb B7/21 which recognizes a monomorphic determinant on HLA-DP (21) and was used and validated also in the previous study by Thomas et al. (5) (Figure 1A). Compared with rs9277534-A/A (AA), BLCL homozygous for rs9277534-G/G (GG) showed significantly higher amounts of HLA-DP molecules, while no difference was observed in HLA-DR expression (Figure 1A, Table 4). On primary B cells, we confirmed the original finding of Thomas et al. with expression levels of HLA-DP significantly higher in primary unstimulated B cells from healthy blood donors homozygous for GG in comparison to AA, while no difference was observed in HLA-DR expression (Figure 1B, Table 4). Interestingly, overnight incubation with IFN-γ to simulate inflammatory conditions abrogated these differences (Figure 1C, Table 4).

**Table 4:** HLA-DP and HLA-DR cell surface expression in different cell types.

		**HLA-DP**^**c**^		**HLA-DR**^**c**^	
**APC^a^**	**rs9277534 (N)^b^**	**mean±SD**	***p***	**mean±SD**	***p***
BLCL	AA (8)	2.45 × 10^5^ ± 1.27	**	7.48 × 10^5^ ± 2.06	n.s.
	GG (9)	5.22 × 10^5^ ± 1.85		8.62 × 10^5^ ± 1.44	
B cell	AA (11)	0.32 × 10^5^ ± 0.15	*	1.64 × 10^5^ ± 0.69	n.s.
	GG (11)	0.50 × 10^5^ ± 0.25		1.40 × 10^5^ ± 0.67	
B cells + IFN-γ^d^	AA (11)	0.44 × 10^5^ ± 0.21	n.s.	1.82 × 10^5^ ± 0.72	n.s.
	GG (11)	0.68 × 10^5^ ± 0.40		1.64 × 10^5^ ± 0.82	
Monocytes	AA (11)	0.17 × 10^*e*^ ± 0.17	*	1.61 × 10^5^ ± 1.35	n.s.
	GG (11)	0.37 × 10^5^ ± 0.26		1.83 × 10^5^ ± 1.26	
Monocytes + IFN-γ^e^	AA (11)	1.02 × 10^5^ ± 0.37	n.s.	4.46 × 10^5^ ± 1.02	n.s.
	GG (11)	1.31 × 10^5^ ± 0.58		3.81 × 10^5^ ± 1.29	
Immature DC	AA (10)	1.23 × 10^5^ ± 0.95	n.s.	3.73 × 10^5^ ± 2.60	n.s.
	GG (10)	2.71 × 10^5^ ± 2.20		3.84 × 10^5^ ± 2.78	
Mature DC	AA (10)	5.56 × 10^5^ ± 2.71	n.s.	10.62 × 10^5^ ± 2.71	n.s.
	GG (10)	6.86 × 10^5^ ± 5.26		8.36 × 10^5^ ± 3.69	

a *HLA-DP and HLA-DR expression was quantified by flow cytometry on the cell surface of different cell types as described in Materials and Methods. Lists of the APC used in this study are reported in **Table 1,2***.

b *rs9277534-G/A as determined locally*.

c *Cell surface expression of HLA-DP and HLA-DR are reported as converted MEPE values as described in Materials and Methods*.

d *IFN-γ significantly upregulated HLA-DP and DR expression on total B cells: HLA-DP untreated 0.41x10^5^ ± 0.22 vs. IFN-γ treated 0.56x10^5^ ±0.34 (p < 0.0001); HLA-DR untreated 1.51x10^5^ ± 0.67 vs. IFN-γ treated 1.73x10^5^ ±0.76 (p < 0.001)*.

e IFN-γ significantly upregulated HLA-DP and DR expression on total monocytes: HLA-DP untreated 0.27x10^5^ ± 0.24 vs. IFN-γ treated 1.17x10^5^ ±0.50 (p < 0.0001); HLA-DR untreated 1.72x10^5^ ± 1.28 vs. IFN-γ treated 4.14x10^5^ ±1.18 (p < 0.0001). Statistical comparisons were performed by two-tailed unpaired t-test (n.s. not significant;

* p < 0.05;

** *p < 0.01)*.

**Figure 2:**
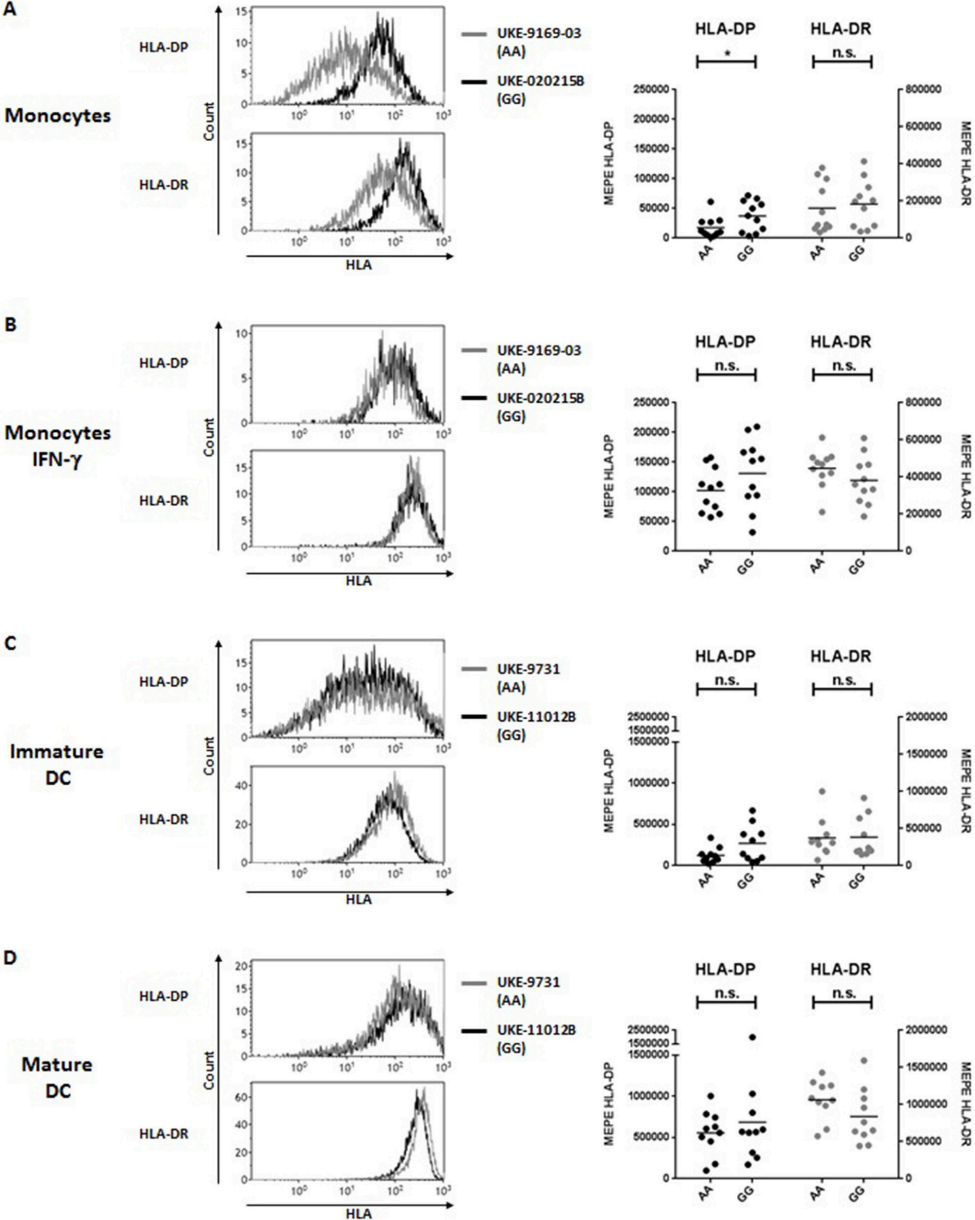
HLA-DP and HLA-DR cell surface expression in relation with rs9277534 SNP in monocytes and DC. Cell surface staining for HLA-DP and HLA-DR was performed with the mAb B7/21 and L243, respectively, on **(A,B)** CD14+ monocytes in PBMC after 48 h incubation with medium (*N* = 22) (**A**) or with 200 IU/ml IFN-γ (*N* = 22) (**B**), and on **(C)** monocyte-derived immature DC (*N* = 20) or **(D)** mature DC (*N* = 20). Cells were homozygous for either rs9277534-A/A (AA) or rs9277534-G/G (GG) (Table 2). Shown are two representative FACS plots (left panel) and the Molecules of Equivalent Soluble PE (MEPE) of all samples (right panels). Statistical comparisons were performed by the two-tailed unpaired *t*-test (n.s. not significant; *p < 0.05). Mean ± SD are reported in Table 4.

To address the question whether these observations also apply to other APC potentially relevant in allogeneic HSCT, we carried out similar analyses in primary monocytes and monocyte-derived DC. Similar to primary B cells, significant differences for HLA-DP but not HLA-DR expression were found in primary monocytes between individuals homozygous for rs9277534-A/A or rs9277534-G/G (Figure 2A, Table 4), but these differences were also abrogated after overnight incubation with IFN-γ (Figure 2B, Table 4). Importantly, no significant differences for either HLA-DP or HLA-DR expression were observed in association with rs9277534 for immature DC (Figure 2C, Table 4) or mature DC (Figure 2D, Table 4).

### Genetic control of HLA-DPB1 transcriptional levels in BLCL and DC

**Figure 3:**
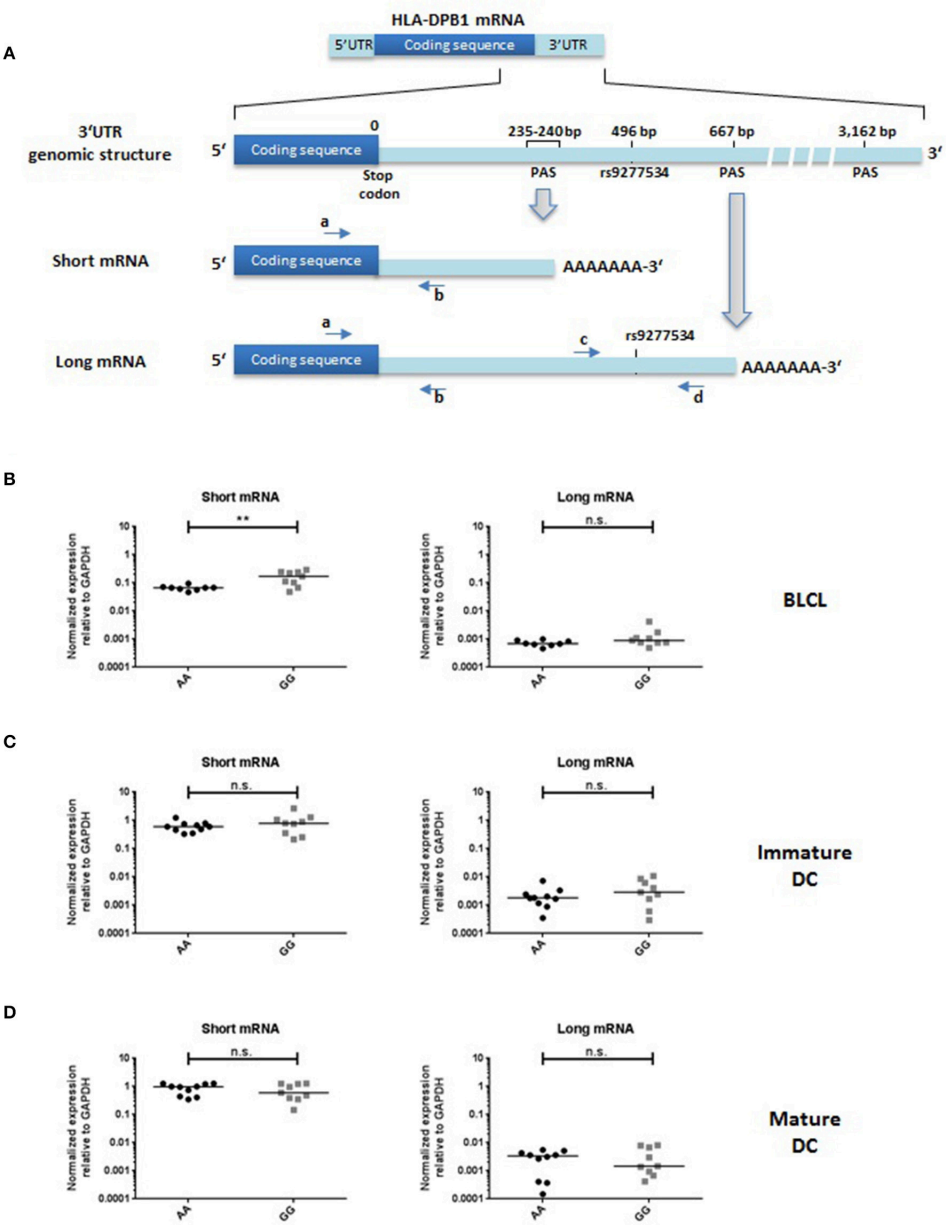
HLA-DPB1 transcriptional expression in relation with rs9277534 SNP in BLCL and DC. **(A)** Schematic representation of the HLA-DPB1 genomic structure and a short and long form of mRNA generated by two different polyadenylation sites (PAS) about 400 bp apart, as reported in the GenBank database (ID: NM_002121.5). Note that the long but not the short mRNA includes the rs9277534 SNP. Primer locations for qPCR quantification of both transcripts are indicated as arrows a-b or c-d (see also Table 3). Primers “a” and “b” anneal to both the short and the long mRNA, while primers “c” and “d” are specific for the long mRNA. **(B–D)** Short (left panel) and long (right panel) HLA-DPB1 mRNA was quantified in BLCL (*N* = 17, **B**), immature DC (*N* = 20, **C**) and mature DC (*N* = 20, **D**), by qPCR relative to GAPDH as described in Materials and Methods. Cells were homozygous for either rs9277534-A/A (AA) or rs9277534-G/G (GG) (Tables 1,2). Statistical comparisons were performed by the two-tailed unpaired *t*-test (n.s. not significant; **p < 0.01).

The HLA-DPB1 3′UTR is over 3000 bp in length and contains three sets of PAS, located at position 235–240, 667, and 3162, respectively (GenBank ID: NM_002121.5). The rs9277534 SNP is located at position 496 between the first and the second PAS which mediate the synthesis of at least two mRNA isoforms of different length, a short one using the more proximal and a long one using the more distal PAS. The rs9277534 SNP is contained in the long but not in the short transcript (Figure 3A). Transcriptional expression levels of HLA-DPB1 were comparatively determined for the short and the long transcript by qPCR in BLCL, immature and mature DC. Consistent with previous reports (5, 8) and with our findings on HLA-DP protein expression levels, BLCL homozygous for rs9277534-G/G carried significantly higher transcript levels of the short mRNA isoform lacking the rs9277534 SNP, compared with rs9277534-A/A (mean normalized expression relative to GAPDH was 0.1666 ± 0.08686 vs. 0.0665 ± 0.01387, *p* < 0.01. Transcription levels of the long mRNA isoform including the rs9277534 SNP were at least 2-log lower compared with the short, and not significantly different between the two groups of BLCL (mean normalized expression relative to GAPDH 0,0013 ± 0.00114 vs. 0.0007 ± 0.00017, *p* = 0.18; Figure 3B). This observation suggests that the rs9277534 SNP probably does not have a direct role in regulating mRNA transcript levels in BLCL, but that rather other, linked polymorphisms could mediate this function. Consistent with our findings on protein expression levels, no significant differences in the transcript levels of either the short or the long mRNA isoform were observed in immature and mature DC from individuals homozygous for rs9277534-G/G or rs9277534-A/A (Figures 3C,D).

### HLA-DPB1 3′UTR haplotypes

**Figure 4:**
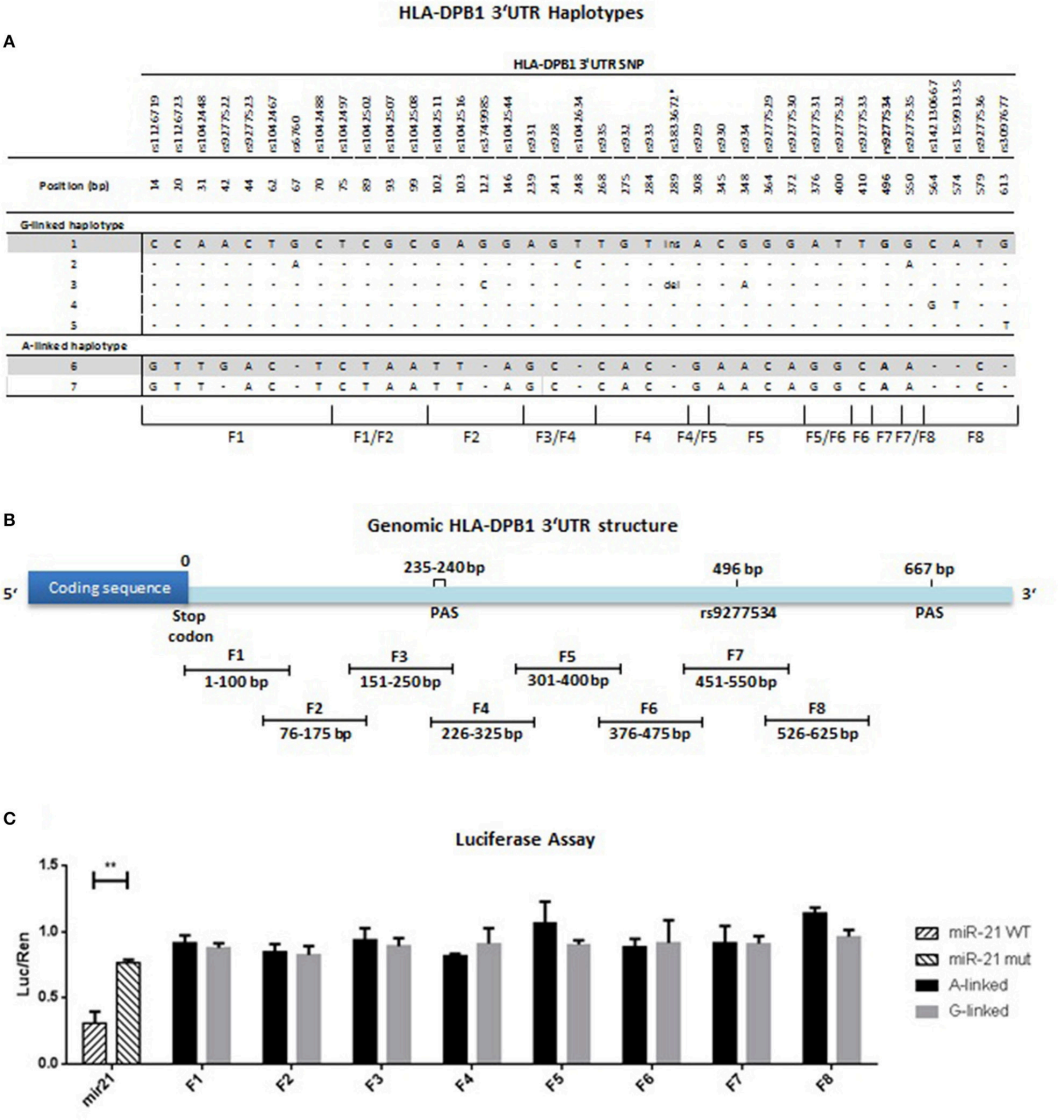
HLA-DPB1 3′UTR haplotype identification, mapping and regulatory activity. **(A)** 671 bp of the 3′UTR (i.e., from the last 4 bp of exon 5 to the first 667 bp of exon 6) of 30 HLA-DPB1 alleles reported as common in Europe (Table 5) (27) were aligned from the IMGT/HLA database (23). Nucleotide polymorphism was restricted to 37 bi-allelic SNP and one insertion/deletion (ins/del), and is listed with the respective position relative to the stop codon in exon 5. The rs9277534-G/A SNP located at position 496 is indicated in bold. A total of 7 haplotypes were identified, 5 linked to rs9277534-G and 2 linked to rs9277534-A. Nucleotide polymorphism is indicated with reference to the rs9277534-G linked haplotype 1, dashes indicate sequence identity. Haplotypes 1 and 6 were chosen for further studies and are highlighted in gray. **(B)** Mapping of the HLA-DPB1 3′UTR. Eight overlapping fragments F1-F8, each 100 bp in length with an overlap of 25 bp, were PCR-amplified from haplotype 1 (BLCL AKIBA, HLA-DPB1*09:01) or haplotype 6 (BLCL MOU, HLA-DPB1*04:01) and cloned into the pmirGLO dual luciferase vector as described in Materials and Methods. Note that fragment F7 comprises rs9277534. **(C)** Gene regulation by HLA-DPB1 3′UTR fragments assessed with the dual luciferase assay. Four different BLCL (APA, MOU, BM21, OSR-6924) were transfected with pmirGLO containing F1-F8 from haplotype 1 (rs9277534-G linked, gray bars) or from haplotype 6 (rs9277534-A linked, black bars), respectively, and tested in 3 independent experiments by dual luciferase assays as described in Materials and Methods. A total of 12 independent analyses were performed for each fragment. 50 bp fragments containing the miR21 WT or mutated target sequence were used as positive and negative control, respectively (striped bars). Results are expressed as mean of fold-change of luciferase activity respect to renilla (luc/ren). Bars refer to SD between 12 independent experiments. Statistical comparisons were performed by the Wilcoxon matched-pairs signed rank Test (**p < 0.001).

Polymorphic variation in HLA-DPB1 is characterized by tight LD which encompasses the entire genomic region from intron 2 to the 3′UTR (26). We aligned HLA-DPB1 3′UTR sequences from common alleles identified in Europe (27) deposited in the IMGT/HLA database (23) and found a total of 37 SNP, which gave rise to a total of 7 different haplotypes, 5 linked to rs9277534-G and 2 linked to rs9277534-A (Figure 4A and Table 5). The 5 rs9277534-G linked haplotypes 1-5 differ only by 1-3/37 SNP, while at least 28/37 SNP differences are found in the 2 rs9277534-A linked haplotypes 6 and 7, which in turn are strikingly similar to each other. Interestingly, the haplotypes are in tight LD with a STR located in intron 2 at 44 bp upstream of exon 3, with G- and A-haplotypes linked with a short and a long STR, respectively (Table 5). Moreover, LD also encompasses the exon sequences, with TCE groups 1 and 2 linked with the G-haplotype and TCE group 3 linked with the A-haplotype, although a number of exceptions do exist (Table 5).

**Table 5:** HLA-DPB1 3′UTR haplotypes identified in this study.

		HLA-DPB1^*^			
**3′UTR Haplotype**	**Frequency^a^**	**Allele^b^**	**TCE group^c^**	**Expression SNP^d^**	**Intron 2 STR^e^**
1	0.191	03:01^f^	2	G	(GGAA)_4_
		05:01	3		
		06:01	2		
		09:01^f^	1		
		10:01^f^	1		
		14:01	2		
		15:01^f^	3		
		45:01	2		
		63:01	3		
		130:01	3		
2	0.062	01:01^f^	3		
		26:01	3		
3	0.045	11:01	3		
		13:01^f^	3		
4	0.006	19:01	2		
		34:01	3		
5	n.o.	18:01	3		
6	0.677	02:01^f^	3	A	(GGAA)_10−16_
		02:02	3		
		04:01^f^	3		
		04:02^f^	3		
		17:01	1		
		23:01	3		
		41:01	3		
		46:01	3		
		47:01	3		
		105:01	3		
		124:01	2		
		126:01	3		
7	n.o.	30:01	1		

a *Combined frequency of HLA-DPB1 alleles as reported in www.allelefrequencies.net database (England Northwest population, N = 2960), n.o. not observed*.

b *Listed are only those HLA-DPB1 alleles reported to be common in Europe (27)*.

c *TCE group assigned according to Crivello et al (15)*.

d *Linked allelic variant at SNP rs9277534 associated with high (G) or low (A) HLA-DPB1 expression (8)*.

e *Number of repeated units of the STR (AAGG)n in Intron 2 of HLA-DPB1 as reported in IMGT/HLA databank (Release 3.31.0 2018-01, http://www.ebi.ac.uk/ipd/imgt/hla)*.

f *For this allele, the 3′UTR sequence was confirmed by direct sequencing on a homozygous BLCL as listed in Table 2*.

### Mapping of regulatory elements in the 3′UTR of HLA-DPB1 by luciferase assays

In order to determine whether the rs9277534 itself and/or other linked SNP might be directly involved in differential regulation of transcript abundance, the HLA-DPB1 3′UTR was mapped into a total of eight overlapping fragments of 100 bp each, covering the polymorphisms in the region from the stop codon to the second PAS at position 667, including rs9277534 in fragment F7 (Figure 4B). Fragments F1-F8 from haplotypes 1 and 6, prototypes of rs9277534-G and rs9277534-A haplotypes, respectively (Figure 4A and Table 5), were cloned downstream of a luciferase reporter gene and tested by standard luciferase assays in 4 different BLCL, a cell type in which the genetic control of HLA-DPB1 transcript levels by the rs9277534 SNP was evident. Significant differences in luciferase activity were observed between mir21-WT and mir21-mut used as positive and negative controls, respectively (Figure 4C). Compared with the positive control, none of the eight fragments, including F7 encompassing rs9277534, was able to markedly reduce luciferase activity. Moreover and importantly, no significant differences were observed when comparing luciferase activity under the control of the same fragment cloned from haplotype 1 or from haplotype 6 (Figure 4C). Taken together, these results did not define any major regulatory element in the 3′UTR of HLA-DPB1, suggesting that polymorphism in other regions of the gene might be involved.

### Assessment of alternative splicing associated with the HLA-DPB1 intron 2 STR

**Figure 5:**
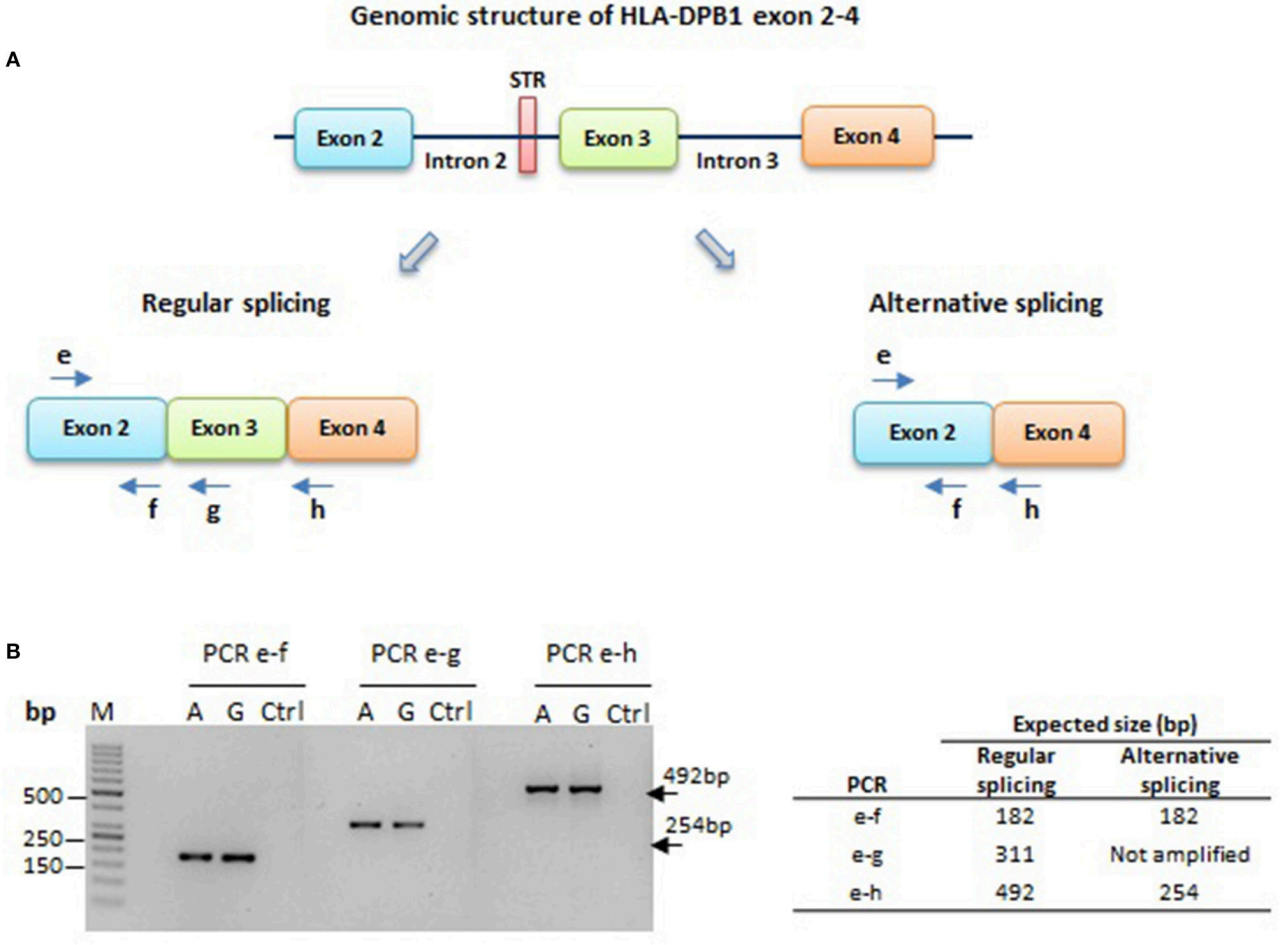
HLA-DPB1 exon 2–4 splicing in HLA-DPB1 rs9277534-G/A haplotypes. **(A)** A short tandem repeat (STR) of 4 or 10-16 GGAA repeats associated with the rs9277534-G or -A SNP, respectively, is located 44 bp upstream of exon 3 (Table 5) (28). The longer STR variant might facilitate splicing of exon 3, giving rise to an alternative splice product joining exons 2 and 4, which would not produce a functional HLA-DP protein. To test this hypothesis, PCR amplicons were generated using a common forward primer “e” in exon 2, and 3 reverse primers “f”, “g” and “h” in exon 2, 3 and 4, respectively. **(B)** RT-PCR on RNA from 6 BLCL carrying the rs9277534-G or -A linked haplotypes 1 and 6, respectively (Table 4). Shown is agarose gel electrophoresis obtained with primer pairs e-f, e-g and e-h on one representative BLCL for each haplotype (A or G). All three PCR products of 182 bp, 311 bp and 492 bp respectively were produced, while the 254 bp expected for alternative splicing was absent in both haplotypes. M; Molecular weight marker; Ctrl; no template control.

We next asked whether the long isoform of the intron 2 STR, in linkage with the low expression rs9277534-A variant (Table 5) (28), could induce a splicing defect involving exon 3, which is in relatively close vicinity to the 3′ end of the STR. The resulting hypothetical alternatively spliced transcript would join exon 2 and exon 4, resulting in a complete loss of function (Figure 5A). To test this hypothesis, we designed PCR primers in exon 2, 3 and 4, which would give rise to PCR products of different sizes in the presence or absence of alternative splicing. In a total of 6 BLCL homozygous for a rs9277534-G or a rs9277534-A haplotype, no evidence for alternative splicing was observed (Figure 5B).

### Expression model vs. TCE structural model of donor-recipient HLA-DPB1 mismatches in HSCT

**Figure 6:**
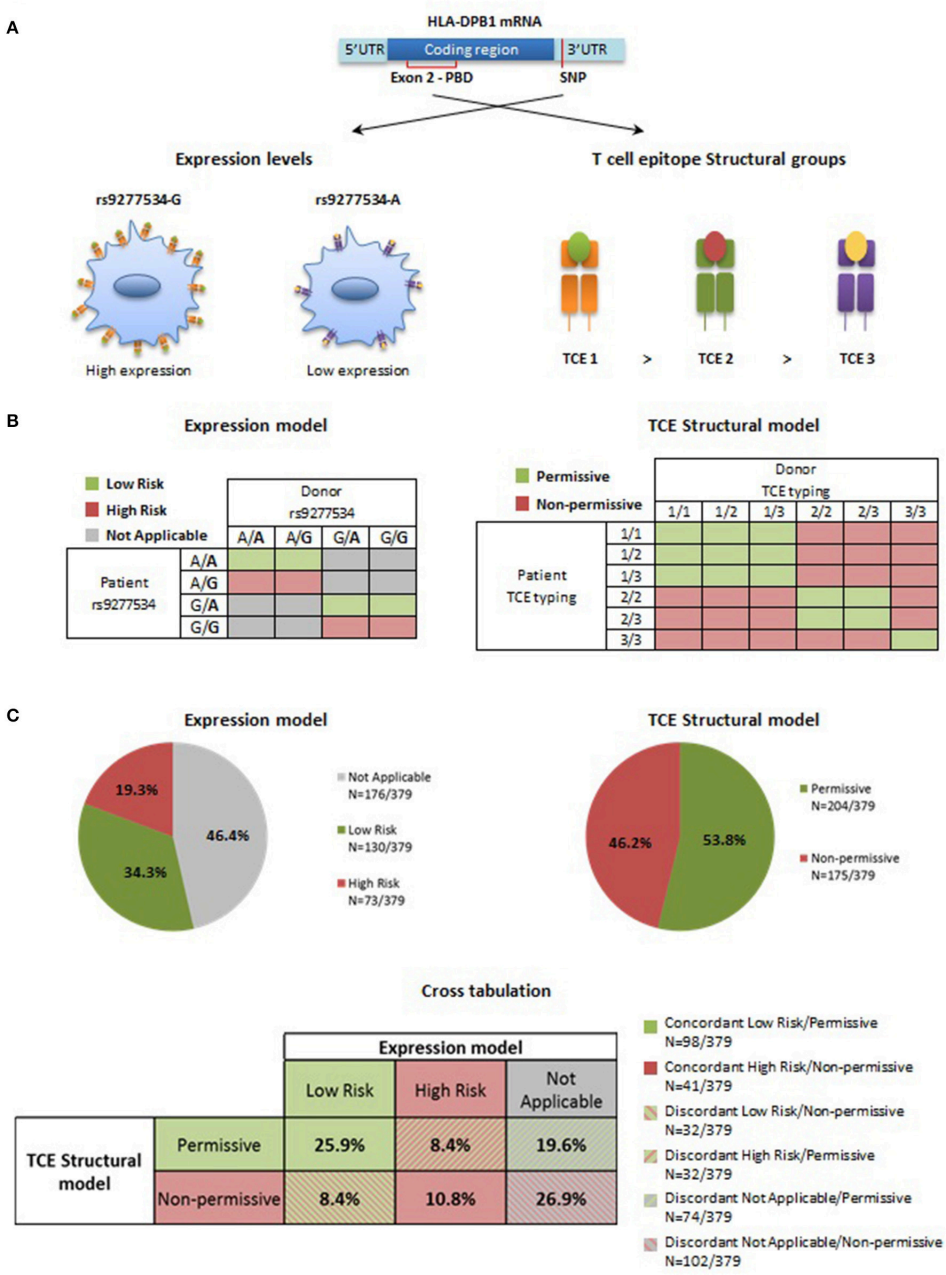
Expression model and TCE Structural model for HLA-DPB1 mismatches in HSCT. **(A)** Genetic control of HLA-DP expression levels is mediated by the biallelic SNP rs9277534, with a high expression G- and a low expression A-variant (5). Three distinct TCE groups with decreasing immunogenicity are determined by structural amino acid variability in the peptide binding domain (PBD) encoded by exon 2 in the HLA-DPB1 coding region (9, 15). **(B)** For Expression matching, only truly single HLA-DPB1 allele mismatches in GvH direction are considered, leading to a number of combinations that cannot be classified (in gray). For the others, favorable (low risk, in green) and unfavorable (high risk, in pink) pairs are those where the mismatched allele in the patient carries the low expression rs9277534-A or the high expression rs9277534-G variant, respectively (8). For TCE Structural matching, favorable (permissive, in green) pairs are those in which patient and donor share at least one HLA-DPB1 allele from the TCE group with the highest immunogenicity; the others are predicted to be unfavorable (non-permissive, in pink) (9, 11). **(C)** Percentage of favorable and unfavorable HLA-DPB1 mismatches according to the Expression model or the TCE Structural model in 379 donor-recipient pairs from the University Hospital Essen. Pies indicate the percentage of favorable (low risk or permissive, in green) or unfavorable (high risk or non-permissive, in pink) combinations according to the two models, or of pairs that cannot be classified by the Expression model (not applicable, gray). Classification by the two models has a 36.7% concordance (filled boxes in the cross-tabulation), and 63.3% discordances (striped boxes in the cross-tabulation), either due to differential risk assignment (background green or pink) or to non-applicability of the Expression model (background gray).

Two currently proposed HLA-DPB1 mismatch models, i.e., the Expression model and the TCE Structural model (**Figure 6A**), take the classical concept of donor-recipient matching for polymorphic HLA-DPB1 alleles further to matching for HLA-DPB1 allele groups. For the Expression model, each of the two HLA-DPB1 alleles in patient and donor is assigned to the rs9277534-G or –A group, based on tight LD between this SNP and the different alleles (8). The Expression model is considered to be predictive only for single HLA-DPB1 mismatches in GvH direction, i.e., recipient and donor are matched for the HLA-DPB1 allele on one haplotype but mismatched for the other. These single mismatches are classified as unfavorable (“high-risk”) if there is a patient-specific HLA-DPB1 allele not shared by the donor which belongs to the high expression rs9277534-G group, and as favorable (“low risk”) if it belongs to the low expression rs9277534-A group. All other combinations, including those in which there is no patient-specific single mismatched allele, or in which patient and donor are mismatched for HLA-DPB1 alleles on both haplotypes, cannot be classified as favorable or unfavorable by the Expression model (“not applicable”; Figure 6B, left panel). For the TCE Structural model, each of the two HLA-DPB1 alleles in patient and donor is assigned to one of the three TCE groups, and mismatches are considered to be unfavorable (“non-permissive”) if the TCE group of the highest order in the hierarchy is not shared between patient and donor, while all other mismatch combinations are considered to be favorable (“permissive”; Figure 6B, right panel).

Risk prediction by the Expression model and the TCE Structural model was comparatively evaluated in 379 HLA-DPB1-mismatched donor-recipient pairs from the University Hospital Essen (29). In the Expression model, 73 (19.3%) and 130 (34.3%) were classified as high or low-risk, respectively, while 176 (46.4%) pairs were “not applicable” because they did not satisfy the pre-requisite of single HLA-DPB1 allele mismatches in GvH direction (Figure 6C, upper left panel). In the TCE Structural model, all 379 (100%) pairs could be classified and of these, 175 (46.2%) and 204 (53.8%) fell into the non-permissive or the permissive group, respectively (Figure 6C, upper right panel).

The overall concordance between the two models was 36.7% with assignment as permissive/low risk in 98/379 (25.9%) and non-permissive/high risk in 41/379 (10.8%) pairs, respectively. 240/379 (63.3%) assignments were discordant. These were due to differential risk prediction in 64/379 (16.8%) pairs, with even distribution between permissive/high risk and non-permissive/low risk, each occurring in 32/379 (8.4%) pairs (Figure 6C, lower panel). The remaining discordances were due to non-applicability of the expression model, occurring in 176/379 (46.5%) pairs. Of these, a slightly higher number (102/176; 58%) was classified as non-permissive, compared to the remaining 74/176 (42%) pairs classified as permissive, corresponding to an overall percentage of 26.9% and 19.6%, respectively, in the cohort overall (Figure 6C, lower panel).

### T-cell alloreactivity to HLA-DP expressed in HeLa-II cells in the absence of the 3′UTR

**Figure 7:**
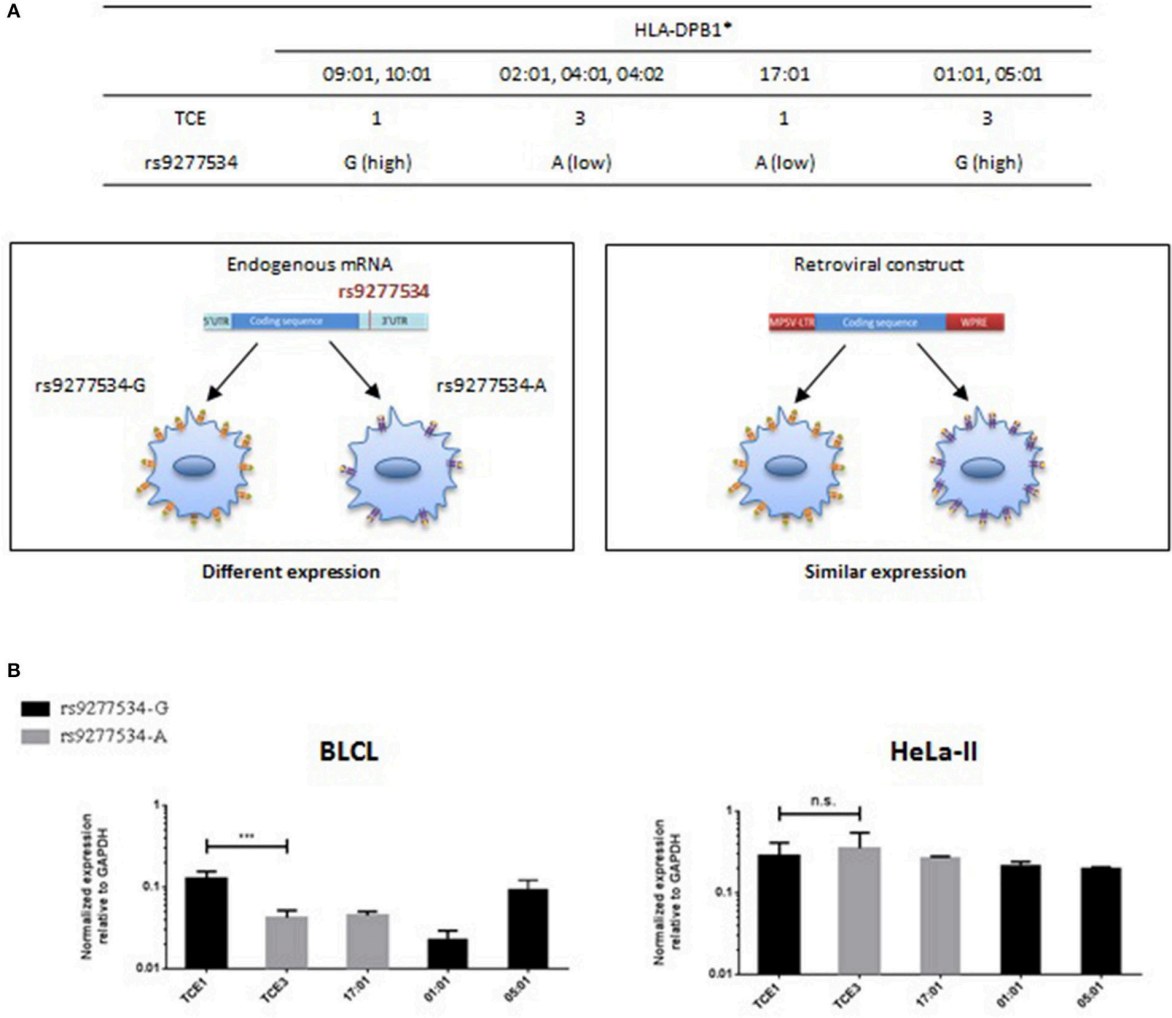
HLA-DPB1 transcriptional expression in BLCL or transduced HeLa-II in relation with rs9277534 and TCE groups. **(A)** The most frequent HLA-DPB1 alleles from TCE group 1 and 3 are linked with rs9277534-G and -A, respectively (16). Notable exceptions are HLA-DPB1*17:01 (TCE 1), HLA-DPB1*01:01 and HLA-DPB1*05:01 (both TCE 3), in which this association is inverted. Endogenous HLA-DPB1 expression is predicted to be genetically regulated by the rs9277534-G/A SNP in the 3′UTR, while this regulation is abrogated in cells transfected with the HLA-DPB1 coding region under a retroviral promoter in the absence of the 3′UTR. **(B)** Transcriptional expression of HLA-DPB1 in BLCL or transduced HeLa-II. HLA-DPB1 mRNA was quantified by RT-qPCR relative to GAPDH in BLCL homozygous for alleles from TCE 1 (rs9277534-G) or TCE 3 (rs9277534-A) (Table 1), or in HeLa-II after retroviral vector transduction with the same alleles. Shown are the mean results and SD from 3 independent experiments. Statistical comparisons were performed by the two-tailed unpaired *t*-test (***p < 0.001).

The most frequent HLA-DPB1 alleles in rs9277534-G and rs9277534-A haplotypes belong to TCE groups 1/2 and TCE group 3, respectively (Table 5). However, a few exceptions exist, in particular HLA-DPB1^*^17:01 from TCE group 1, which is associated with the rs9277534-A haplotype 6, and HLA-DPB1^*^01:01 and ^*^05:01, both of which are found in linkage with the rs9277534-G haplotypes 2 and 1, respectively (Table 5 and Figure 7A). We expressed 5 HLA-DPB1 alleles representative of the “correct” association (HLA-DPB1^*^09:01, ^*^10:01 – TCE group 1 and rs9277534-G; HLA-DPB1^*^02:01, ^*^04:01, ^*^04:02 – TCE group 3 and rs9277534-A), as well as the 3 abovementioned alleles with the “inverted” association in HeLa-II cells, under the control of a retroviral promoter (18), in the absence of the 3′UTR (Figure 7A). As expected, the significant differences observed in BLCL between transcript levels of HLA-DPB1 alleles from TCE group 1 (rs9277534-G) and TCE group 3 (rs9277534-A) were abrogated in transduced HeLa-II cells (Figure 7B).

**Figure 8:**
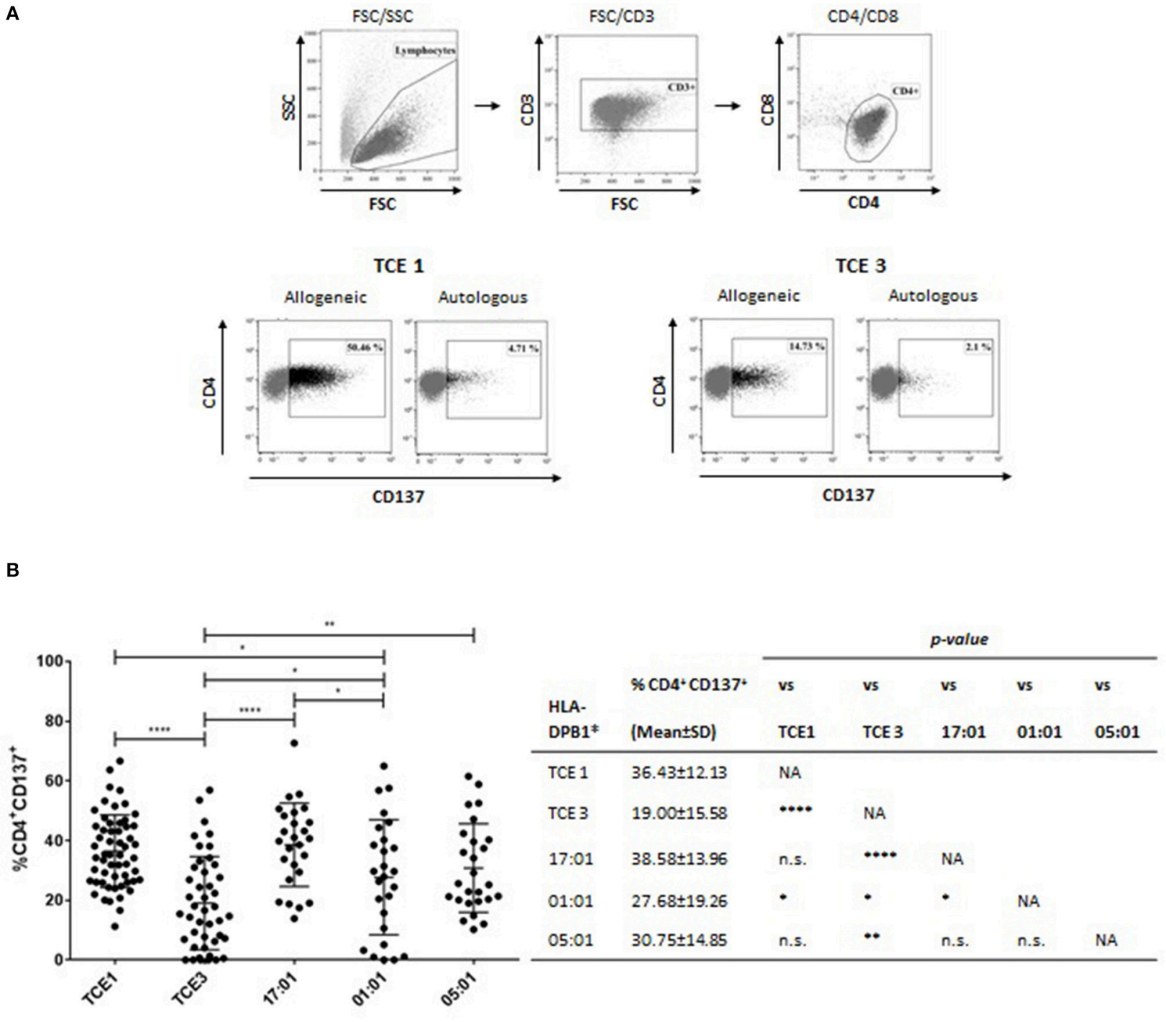
T-cell allorecognition of HLA-DPB1 from different TCE groups expressed by HeLa-II. PBMC from HLA-DPB1 TCE 1-negative responders carrying at least one HLA-DPB1 TCE 3 were stimulated with irradiated HeLa-II transduced with allogeneic HLA-DPB1 TCE 1 or TCE 3 for 14 d, as described in Materials and Methods. For HLA-DPB1 typing of responders see Table 2. Cultures were re-stimulated for 24 h with HeLa-II expressing the original allogeneic HLA-DPB1, or with autologous HLA-DPB1, and the percentage of CD4+ T cells expressing the CD137 activation marker was assayed by flow cytometry. **(A)** Gating strategy and representative FACS plots for re-stimulation with autologous HLA-DPB1 or with allogeneic HLA-DPB1 from TCE 1 or TCE 3. Note the higher percentage of CD137+ CD4+ T cells in response to TCE 1 compared to TCE 3. **(B)** Results of CD137 assays in response to TCE 1, TCE 3 or HLA-DPB1*17:01, *01:01 or *05:01 for a total of 178 independent co-cultures. Statistical comparisons were performed by the two-tailed unpaired *t*-test (*p < 0.05; **p < 0.01; ****p < 0.0001).

The transduced HeLa-II cells were used in a total of 178 independent cultures for a 14-day stimulation of CD4+ T cells from healthy donors expressing at least one HLA-DPB1 allele from TCE group 3 and a second allele from either TCE group 2 or 3, for whom allogeneic HLA-DPB1 alleles from TCE group 1 and TCE group 3 represent a non-permissive and a permissive mismatch, respectively. The alloreactive response was measured after overnight re-challenging with the original target cell, compared to HeLa-II transduced with autologous HLA-DPB1 as background control, as the percentage of CD4+ T cells up-regulating the activation marker CD137, which has been shown to best describe the total pool of alloreactive T cells (30) (Figure 8A). In line with our previous reports (31), the mean alloresponse to HeLa-II transfected with HLA-DPB1 alleles from TCE group 1 (non-permissive mismatch) was significantly higher compared with TCE group 3 (permissive mismatch; 36.43 ± 12.13 vs. 19.00 ± 15.58, *p* < 0.0001, Figure 8B). Consistent with its classification as TCE group 1, the median response to allogeneic HLA-DPB1^*^17:01 was also significantly higher compared with TCE group 3 (38.58 ± 13.96, *p* < 0.0001, Figure 8B), and not significantly different from TCE group 1, despite similar cell surface expression levels (Figure 7B). For HLA-DPB1^*^01:01, responses were highly variable (27.68 ± 19.26) and significantly different from other alleles of both TCE group 1 and TCE group 3 although both with a low level of significance (*p* < 0.05 each). Similar levels of responses were also seen against HLA-DPB1^*^05:01 (30.75 ± 14.85), although these were significantly different (*p* < 0.01) from TCE group 3 but not from TCE group 1 (Figure 8B). These data suggest that HLA-DPB1^*^01:01 and 05:01 appear to represent a functionally distinct TCE group. Taken together, our data demonstrate that also in the absence of significant differences of transcriptional HLA-DPB1 alleles on HeLa-II cells, differential *in vitro* T-cell alloreactivity against permissive and non-permissive mismatches can be appreciated.

## Discussion

Our study provides new insights into the cell type-specific and mechanistic basis of the association between the rs9277534 SNP and HLA-DPB1 expression, and into the relationship between the Expression model and the TCE Structural model for HLA-DPB1 mismatch risk prediction in HSCT.

We are the first to broaden observations on the genetic control of HLA-DPB1 expression from BLCL and primary unstimulated B cells, in which this issue has been studied so far (5, 8), to other cell types likely to play a role in GvHD, such as DC and IFN-γ-stimulated B cells and monocytes. We confirm the previous data from the literature, but also show that the observed differences in HLA-DPB1 cell surface and transcriptional expression levels between the two rs9227534 variants are abrogated after IFN-γ stimulation, and could not be observed in either mature or immature DC. Although a simplistic model, IFN-γ stimulation can be seen as a simulation of an inflammatory environment that can be induced by different triggers such as the conditioning regimen or infection after HSCT, and has been described as one of the most relevant cytokines mediating toxic effects of GvHD after HSCT (32). In addition, DC are crucial APCs for inducing alloresponses particularly from naïve T cells (33, 34). The observation that genetic control of HLA-DP expression by the rs9227534 variation appears to be dampened under these conditions challenges its relevance in the clinical setting. However, it has to be noted that ours are *in vitro* models that may not fully reflect the *in vivo* situation. Moreover and importantly, monocytes and B cells in a non-inflammatory context can have an important role in triggering alloimmunity, in particular from the memory T-cell repertoire, which is involved in direct recognition of major histocompatibility antigen mismatches (35, 36).

We are also the first to attempt a mechanistic understanding of the observed genetic control of HLA-DPB1 expression in BLCL. The data we obtained by 3′UTR mapping and analysis in luciferase assays in BLCL argue against a direct regulatory role of rs9277534 or of any other linked SNP in the 3′UTR. A limitation here is again that this *in vitro* assay might not adequately reflect the *in vivo* situation, which is likely to be considerably more complex, leaving us with the possibility that the interplay of different factors might still lead to a direct SNP regulation of expression. We also did not obtain evidence for alternative splicing associated with the linked intron 2 STR (37). An alternative role of this STR might be transcriptional repression rather than alternative splicing, a possibility we were unable to test due to the unavailability of an appropriate *in vitro* model. In line with observations made by others for HLA-A (2), we show that also the HLA-DPB1 3′UTR contains two alternative PAS giving rise to a short and a long mRNA transcript. In contrast to their observations on HLA-A, however, the long HLA-DPB1 transcript was expressed at markedly lower levels compared to the short one. This further argues against a dominant direct role of the rs9277534 SNP in HLA-DPB1 expression, since this SNP is contained in the long but not in the majorly abundant short mRNA transcript. Nevertheless, a role of the SNP in binding factors involved in determining the stability of the long mRNA, such as described for HLA-A, cannot be ruled out.

Regarding risk prediction by the Expression model and the TCE structural model in HSCT, we show a relatively limited degree of 36.7% concordance in our exemplary cohort, which however was due in the majority of cases to non-applicability of the Expression model. It should be noted that the Expression model requires the presence of a single HLA-DPB1 mismatch in GvH direction, thereby precluding its application in almost half of the pairs under analysis. In 203 pairs where risk prediction could be performed by both models, concordance was 68.5%, with discordances evenly distributed between high risk/permissive and low risk/non-permissive pairs. These data might be useful for clinicians intending to apply either model in unrelated donor searches. The clinical cohort was too small to make any meaningful comparative outcome analysis, and future studies in large well-powered studies are clearly warranted to understand the comparative clinical validity of the two models.

From the experimental side, we confirm and extend our previous observations that non-permissive HLA-DPB1 TCE group mismatches elicit significantly stronger T-cell alloresponses compared to permissive mismatches *in vitro* (31, 38). The observation that this holds true also independently from HLA-DPB1 expression levels is in line with our unpublished evidence that permissiveness of TCE group mismatches is dependent on the HLA-DP peptidome (in preparation). This finding is of practical relevance for the 3 HLA-DPB1 alleles with inverted haplotype association studied here, i.e., HLA-DPB1^*^17:01, ^*^01:01, and ^*^05:01, which together have a 8.61% frequency in Europeans (39). While our data confirm the assignment to TCE group 1 of HLA-DPB1^*^17:01, they suggest that HLA-DPB1^*^01:01 and ^*^05:01 belong to a yet distinct functional TCE group. Interestingly, it has recently been suggested that HLA-DPB1 alleles could be divided into two supertypes having HLA-DP2 and DP5 as respective prototypes (40). This division was based on the allele frequency profile of the Japanese population, and was correlated with the incidence of acute GvHD after unrelated HSCT. The HLA-DP2 and DP5 supertypes closely resemble our TCE group 3 and TCE group 1/2, respectively, with the main exception being HLA-DP5. The data from the present study corroborate the functional distinctness of HLA-DP5, despite its FD score of 2.97 which is clearly compatible with its assignment to TCE group 3 (FD score >2.00) (15). Based on these considerations, and on our unpublished findings on the role of peptides in determining HLA-DP permissiveness, we suggest that TCE group 3, which was coined to accommodate all alleles not included in TCE groups 1 and 2, might be more heterogeneous than expected. In line with this, we have previously suggested that HLA-DP2 might constitute a separate TCE4 group, and shown clinical associations equal to or in some cohorts superior to the 3-group TCE algorithm with clinical outcome (10, 11). Further studies are clearly needed to verify this important point.

In conclusion, the data from the present study suggest that genetic control of HLA-DPB1 expression is cell-type dependent and dampened by inflammatory conditions. While we failed to identify a mechanism for the observed association of HLA-DPB1 expression levels with rs9277534 SNP variation in certain cell types under steady state conditions, our data argue against a direct role of this SNP and in favor of indirect mechanisms mediated by linked polymorphisms. The data on clinical risk prediction by the Expression model and the TCE structural model, as well as those on significant modulation of *in vitro* T cell alloreactivity by HLA-DPB1 TCE groups in the presence of similar transcriptional expression levels, suggest that the two models are partially overlapping yet largely distinct. These findings are of both biological and potentially clinical relevance for investigators interested in unrelated stem cell donor selection.

## Ethics statement

This study was carried out in accordance with the recommendations of University Hospital Essen with written informed consent from all subjects in accordance with the Declaration of Helsinki. The protocol was approved by the local Ethics Committee of University Hospital Essen.

## Author contributions

PC, TM, and KF designed the study. TM, EA-B, MM, M-ML, and PC performed experiments. PvB and JF produced the transduced HeLa cells; DB contributed HLA typings of the transplant cohort. PH contributed PBMC from healthy blood donors and their HLA typing. PC and KF wrote the manuscript. TM and EA-B proof-read the manuscript.

### Conflict of interest statement

The authors declare that the research was conducted in the absence of any commercial or financial relationships that could be construed as a potential conflict of interest.
